# Aprepitant alleviates acute lung injury in a rat model of hepatic ischemia–reperfusion via NLRP3/IL-1β signaling pathway

**DOI:** 10.1038/s41598-025-25930-4

**Published:** 2025-11-18

**Authors:** Walaa Yehia Abdelzaher, Mina T. Kelleni, Marly Nady Adly, Mina Ezzat Attya, Michael Atef Fawzy, Mohamed A. Ibrahim

**Affiliations:** 1 Medical Pharmacology Department, Faculty of Medicine, Minia University, Minia, 61511 Egypt; 2 Faculty of Nursing, Lotus University, Minia, 61768 Egypt; 3 Department of Pathology, Faculty of Medicine, Minia University, Minia, 61511 Egypt; 4 Department of Biochemistry, Faculty of Pharmacy, Minia University, Minia, 61511 Egypt

**Keywords:** Aprepitant, Hepatic ischemia reperfusion, Acute lung injury, NLRP3, IL-1β, Gastrointestinal diseases, Respiratory tract diseases

## Abstract

Hepatic ischemia reperfusion (HIR) injury is a complication that complicates major liver surgeries and contributes to significant hepatic and remote organs damage. Aprepitant (Ap), a neurokinin-1 receptor (NK-1R) antagonists, is an antiemetic commonly used in preventing chemotherapy-induced nausea and vomiting. This study aimed to evaluate the potential protective effect of Ap against acute lung injury (ALI) associated with HIR, utilizing the Pringle maneuver to induce 30 min of hepatic ischemia followed by 1 h of reperfusion, while targeting the NLRP3/IL-1β signaling pathway. Serum alanine transaminase (ALT), aspartate transaminase (AST), Lung malondialdehyde (MDA), total antioxidant capacity (TAC), reduced glutathione (GSH), tumor necrosis factor-alpha (TNF-α), caspase-3 levels, NOD-, LRR- and pyrin domain-containing protein 3 (NLRP3) inflammasome, cleaved caspase-3 expressions were evaluated. Hepatic and lung specimens were evaluated histopathologically and an immunohistochemical study of lung interlukin 1beta (IL-1β) was also performed. HIR caused hepatic and lung damage as shown histopathologically and by an increase in serum ALT, AST and lung IL-1β. A significant increase in lung MDA, TNF-α, caspase-3 levels, NLRP3 and cleaved caspase-3 expressions and decrease in TAC and GSH parameters were detected. Ap significantly ameliorated the oxidative stress, inflammatory, and apoptotic parameters, and this was accompanied by a significant improvement in the histopathological findings with reduction in lung IL-1β. Targeting the NLRP3/IL-1β signaling pathway, as shown by Ap in our murine model, could reveal a promising therapeutic approach to protect against ALI during major liver surgeries.

**Supplementary Information:**

The online version contains supplementary material available at 10.1038/s41598-025-25930-4.

## Introduction

Hepatic ischemia reperfusion (HIR) injury is a common and almost inevitable consequence which complicates several major liver surgeries such as transplantation and resection. It accounts for about 10% of early liver graft failure and it exacerbates both acute and chronic graft rejection resulting in graft dysfunction and multiple remote organ damage such as in the lungs, kidneys, heart and brain^1–3^.

There have been reports of acute lung injury (ALI) due to HIR in both research studies and experimental animal models^4,5^. Oxidative stress caused by HIR has been repeatedly demonstrated as major factor in the development of lung damage^6^. Abundant reactive oxygen species (ROS) and inflammatory mediators are released from activated Kupffer cells into the systemic circulation causing distant lung injury following HIR^7^. NOD-, LRR- and pyrin domain-containing protein 3 (NLRP3) inflammasome/interleukin-1beta (IL-1 β) pathway is one of most important signaling pathways contributing to ischemia reperfusion (IR)-induced injuries. Inflammasomes are a type of protein complex that enhance immune responses and cause cellular damage. Several inflammasomes were identified e.g., NLRP3, NLRP1, AIM2, and NLRC4. NLRP3 regulates caspase1 activation with maturation of pro-inflammatory cytokines such as IL-1β and IL-18^8^. NLRP3 has a critical role in ALI as it acts as an important catalyst and accelerator of in different lung injuries. Thus, inhibition of NLRP3 stimulation can help in mitigating lung injury^9^.

Neurokinin-1 receptor (NK-1R) antagonists are antiemetic drugs with unique anxiolytic, antidepressant, and antiemetic properties. Aprepitant (Ap) is considered one of NK-1R antagonists. It is approved for the treatment of chemotherapy- induced nausea and vomiting. Ap produces this action by blocking the actions of substance P (SP), which elevates ROS production causing oxidative stress. The high affinity SP neuropeptide NK-1R is expressed on many cells, e.g. neurons, epithelial cells, adipocytes, and immune cells^10,11^.

Importantly, remote organ injury is a critical medical condition that requires effective and preventative strategies to be managed after HIR. Medication that scavenges ROS and reduces inflammation may be helpful^12^. Therefore, based on Ap beneficial effects in minimizing cisplatin-induced hepatic and renal toxicity^13^, and doxorubicin-induced heart damage^14^ due to its anti-inflammatory, anti-oxidant properties**,** anti-apoptotic and antifibrotic effects^15–17^, we aimed for the first time, to the best of our knowledge; to assess the potential protective effect of Ap against HIR- induced lung injury while testing its ability to modulate the crucial NLRP3/IL-1β signaling pathway.

## Material and methods

### Chemicals and drugs

Ap was obtained from Merck sharp and Dohme, UK. Ketamine and xylazine were acquired from Elice pharma, Pakistan and Adwia, Egypt, respectively.

### Animals and experimental design

Wistar albino adult male rats (n = 48) weighing 180–240 g were used in the experimental study. From El-Nahda University (Beni-Suef, Egypt), rats were obtained. Prior to being used in the experiments, the rats were given a conventional diet of commercial rat chow and water from the tap and given two weeks to adapt to their environment. The National Institutes of Health’s guide for the care and use of laboratory animals was followed during the research. A Permission was received from the Study Ethics Committee of the Faculty of Medicine, Minia University (Approval number: **585/2022**) to the protocol of our experiment, and all methods were done in accordance with the relevant scientific guidelines and regulations. The current study also complies with ARRIVE guidelines.

Forty-eight adult male Wister albino rats were divided into 6 groups (8 rats each) as following (Table 1):

**Table 1:** Animal groups.

Group 1(Sham)	Rats received carboxymethylcellulose (CMC) orally with abdominal incision without HIR
Group 2 (Sham + Ap10)	Rats received Aprepitant 10 mg/kg suspended in CMC orally^17^ daily for 5 consecutive days^16^ before abdominal incision without HIR
Group 3 (HIR)	Rats received CMC with laparotomy and occlusion of hepatic blood supply by pringle maneuver for 30 min followed by reperfusion for one hour
Group 4 (Ap5 + HIR)	Rats received Aprepitant 5 mg/kg suspended in CMC orally daily for 5 consecutive days before HIR^18^
Group 5 (Ap10 + HIR)	Rats received Aprepitant 10 mg/kg suspended in CMC orally daily for 5 consecutive days before HIR^18^
Group 6 (Ap20 + HIR)	Rats received Aprepitant 20 mg/kg suspended in CMC orally daily for 5 consecutive days before HIR^18^

All Ap doses were given according to our pilot study and the research made by Qian and Liu^18^ and its duration was according to our pilot study and the research made by Hafez et al.^16^.

### Hepatic ischemia induction

Before the experiment, the rats were fasted for sixteen hours. General anesthesia was used to perform all surgical procedures through injection of ketamine (1 mg/kg) and intraperitoneal Xylazine (0.25 mg/kg)^19^. The rat’ abdomens were incised through midline incision. Hepatic ischemia was induced via Pringle’s maneuver. In this maneuver bulldog clamps were used to perform ischemia by occluding the rats’ portal triad (portal vein, hepatic artery, and common bile duct) completely for 30 min^20^. In order to prevent visceral dehydration, the abdomen was covered by plastic covers and normal saline at 37 °C was injected into the rat’s abdominal cavity. Warming aids (heater and lamps) were used to adjust rats’ temperature at 37 °C by removing the clamps. The abdomen was closed by silk sutures and left for one hour (reperfusion period). The rats’ abdomen was sutured via silk suturing. The rats were sacrificed one hour following reperfusion^21^.

### Blood and tissue sampling

By the end of the experiment the animal scarification was performed by cervical dislocation. Blood samples were gathered from abdominal aorta and centrifuged for 10 min at 4000 × g using centrifuge (Jantezki, T30, and Germany) in order to collect rats’ serum. These sera were stored at − 80 °C for further assessment of various parameters. Lung was divided where part was kept at − 80 °C for the determination of biochemical analysis. Other part of lung was put in 10% formalin for histological examination.

By using homogenizer (Tri-R Stir-R homogenizer, Tri-R Instruments, Inc., Rockville Centre, NY), every one gram of lung tissue was homogenized in 5 ml of phosphate buffer saline (PBS) which prepared by dissolving 0.2 g KCl, 8.01 g NaCl, 0.27 g KH_2_PO_4_ and 1.78 g Na_2_HPO_4_.2H_2_O in 1 litter of distilled water and adjust the PH at 7.4 then this homogenate was centrifuged for 15 min at 4000 × g. The supernatant was taken and stored at − 80 °C for further assessment of various parameters.

### Biochemical analysis

#### Evaluation of total protein in lung tissue

According to the manufacturer’s instructions, total protein concentration was assessed by colorimetric commercial kit (Biodiagnostic Co., Egypt). Catalog No: TP 20 20.

#### Evaluation of lung oxidative stress parameters

Malondialdehyde (MDA) level; a biomarker of lipid peroxidation was tested in lung tissue by MDA kit (Biodiagnostic, Egypt, Catalog No: MD 25 29).

Following the guidelines provided by the manufacturer, total antioxidant capacity (TAC) and reduced glutathione (GSH) were measured using a commercial colorimetric kit (Biodiagnostic, Egypt, Catalog No: TA 25 13; Invitrogen, Thermo Fisher Scientific, Catalog Number EEA020, respectively).

#### Evaluation of inflammatory and apoptotic in lung tissue

Tumor necrosis factor alpha (TNF-α) and caspase-3 levels in lung tissue homogenate were determined by ELISA Kit according to manufacturer’s instructions (SIGMA-ALDRICH, Catalogue Number: RAB0480; Bio vision, Catalog No: E4592-100, respectively).

### Determination of lung NLRP3 and cleaved caspase-3 expressions by Western blotting

After boiling of tissue homogenates (50 μg of total proteins) for 5 min combining loading buffer containing 2‑mercaptoethanol they were applied to 12% sodium dodecyl sulfate- polyacrylamide gel electrophoresis (SDS-PAGE) then running for 2 h at 100 V. After electrophoresis, blotted proteins to polyvinylideneflouride (PVDF) membranes were blocked for 1 h in a Trisbuffered saline (TBS-T) blocking solution containing 5% (w/v) non-fat milk and 0.05% Tween-20. Incubation with primary antibodies Rabbit Anti-NLRP3 and anti-cleaved caspase-3 antibodies (1:1000, Catalog No: ab263899, ab214430, respectively, Abcam, UK), and β-actin (Santa Cruz Biotechnology, Santa Cruz, CA) was allowed overnight at 4 °C. Goat anti-rabbit polyclonal immunoglobulin conjugated with horseradish peroxidase (1:5000) (Cell Signaling Technology Inc., MA, USA) in blocking buffer was used as a secondary antibody. Bands were visualized by chemiluminescence. Protein bands of all groups were quantified densitometrically as fold change relative to the normal control group after being normalized to β-actin using Image J Software.

### Histopathological Procedure and Examination:

The lung tissue specimens of the rats were dissected and preserved in a 10% formalin solution. Then, these tissue specimens were dehydrated in ascending alcohol concentrations, processed and embedded in paraffin. 5 μm thick cross sections were cut using a microtome and placed on glass slides. Furthermore, these tissue sections were subjected to hematoxylin–eosin stain and examined histopathologically by a pathologist who was blinded to different research groups under an Olympus light microscope. The The lung injury was assessed in 10 randomly selected fields (× 200) and was scored as the following score: score 0 represents normal lung with no injury; score 1 represents involvement of less than 25% of the lung by injury; score 2 represents involvement of 25–50% of the lung by injury; score 3 represents involvement of less than 50–75% of the lung by injury and score 4 represents involvement of more than 75% of the lung by injury. Lung injury was recognized as presence of alveolar hemorrhage, alveolar oedema, thickening of the alveolar septa and inflammatory cellular infiltrate^22^.

### Immunohistochemical staining of IL-1β in lung tissue

A formalin fixed paraffin embedded lung tissue blocks were sectioned into 5 µm sections using a microtome. Then, these tissue sections were placed on positively charged glass slides and were immunohistochemically stained manually. Initially, these tissue sections were dewaxed, rehydrated and endogenous peroxidase blocking by immersion in 3% H_2_O_2_ solution for 30 min. Immersion of the slides on sodium citrate puffer (PH = 6) for 2 times 10 min each in a microwave was used for antigen retrieval. Blocking of non-specific staining is performed by treating the slides by UV block. Polyclonal rabbit anti IL-1β antibody (Bioss, USA) was added to each slide at 1:200 dilutions and incubated overnight in the humidity chamber at 4 °C. After that, the biotinylated secondary antibody was added to each slide for 30 min at room temperature. One drop of DAB substrate- chromogen was applied to each slide for 15 min or till the brown colouration was seen. Then, slides were dipped in Mayer’s haematoxylin to be counter stained.

The slides were examined under light microscope magnification × 200 or × 400. IL-1β expression was classified into either positive or negative expression according to the percentage of cells with cytoplasmic IL-1β staining. IL-1β positive expression if ≥ 5% of cells showed cytoplasmic brownish staining while IL-1β expression is considered negative if the expression was in < 5% of cells^23^.

### Statistical analysis

Results were expressed as means ± SEM. Results were analyzed by one-way analysis of variance test (ANOVA) followed by Tukey’s test. Differences with *p* value < 0.05 were considered significant. Graph pad prism was used for statistical analysis (version 7 for windows, GraphPad software, San Diego California USA; www.graphpad.com).

## Results

### Effect of different doses of aprepitant on lung oxidative stress parameters in hepatic ischemia reperfusion-induced acute lung injury in rats

**Fig. 1:**
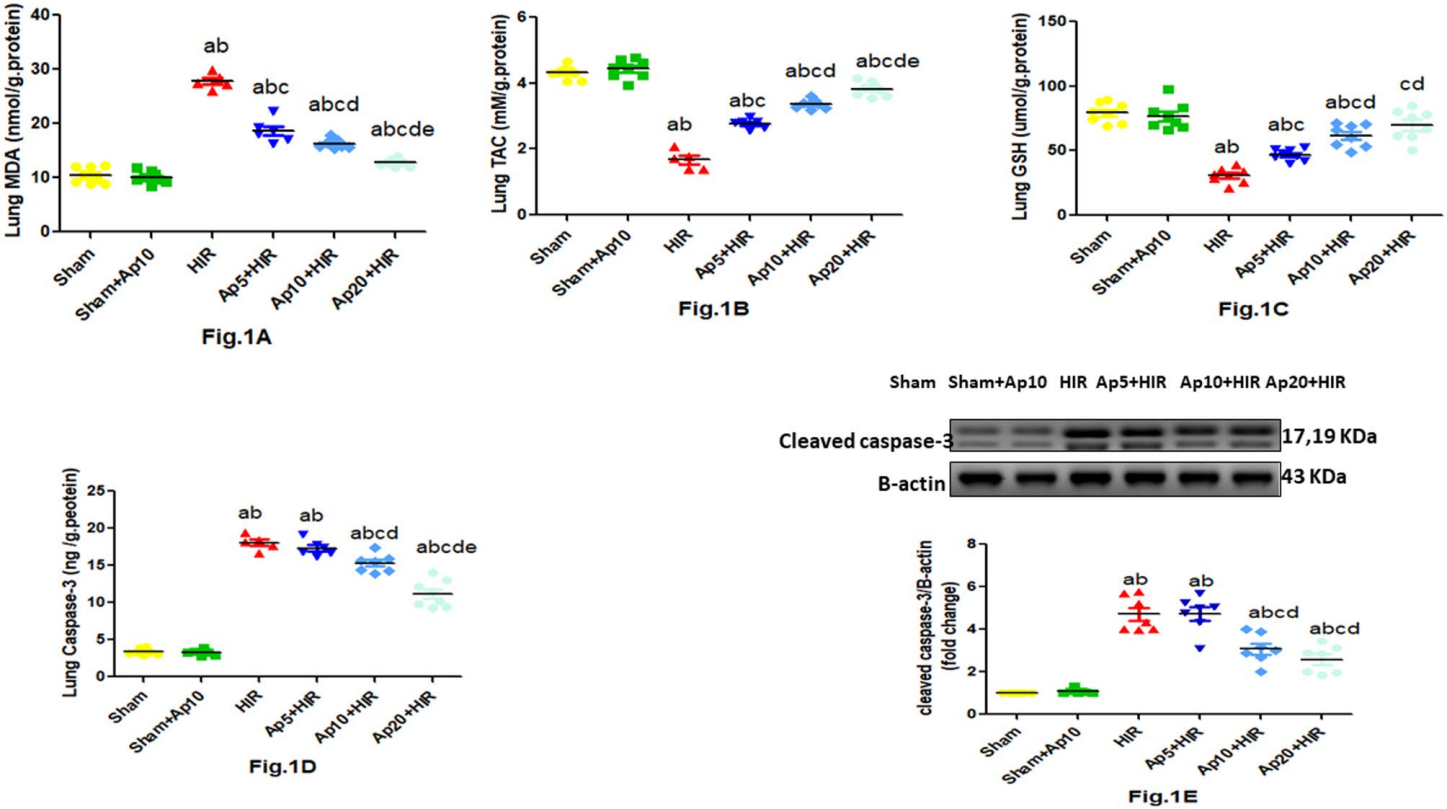
Effect of different doses of aprepitant on lung oxidative stress and apoptotic parameters in hepatic ischemia reperfusion-induced lung injury in rats. Values represent the mean ± SEM (n = 5–8). Results are considered significantly different when *p* < 0.05. ^a^significantly different from sham group. ^b^significantly different from Sham + Ap10 group. ^c^significantly different from HIR group. ^d^significantly different from Ap5 + HIR group. ^e^significantly different from Ap10 + HIR group. (Ap5: Aprepitant 5 mg/kg; Ap10: Aprepitant 10 mg/kg; Ap20: Aprepitant 20 mg/kg; HIR: Hepatic ischemia reperfusion; MDA: Malondialdehyde; TAC: Total antioxidant capacity; GSH: reduced glutathione).

Our study demonstrated a significant increase in levels of lung MDA with a significant decrease in lung TAC and GSH levels in HIR group when compared to sham and Sham-Ap10 groups. Meanwhile administration of Ap5, Ap10 and Ap20 with HIR showed a significant improvement in lung oxidative stress parameters in a dose dependent manner when compared to HIR group (Fig. 1).

### Effect of different doses of aprepitant on lung apoptotic parameters in hepatic ischemia reperfusion-induced acute lung injury in rats

HIR group revealed a significant increase in lung caspase-3 level and cleaved caspase-3 expression in comparison to sham and Sham + Ap10 groups, while administration of Ap10 and Ap20 with HIR showed a significant decrease in level of lung caspase-3 and cleaved caspase-3 expression in a dose dependent manner compared to HIR group (Fig. 1).

### Effect of different doses of aprepitant on lung NLRP-3 inflammasome expression and lung TNF-α level in hepatic ischemia reperfusion-induced acute lung injury in rats

In Fig. 3, HIR showed a significant increase in NLRP-3 expression and lung TNF-α level as compared to sham and Sham + Ap10 groups. On the other hand, Ap5 + HIR, Ap10 + HIR and Ap20 + HIR groups showed a significant decrease in lung NLRP-3 and lung TNF-α level as compared to HIR group.

### Effect of different doses of aprepitant on lung histopathology in hepatic ischemia reperfusion-induced acute lung injury in rats

**Fig. 2:**
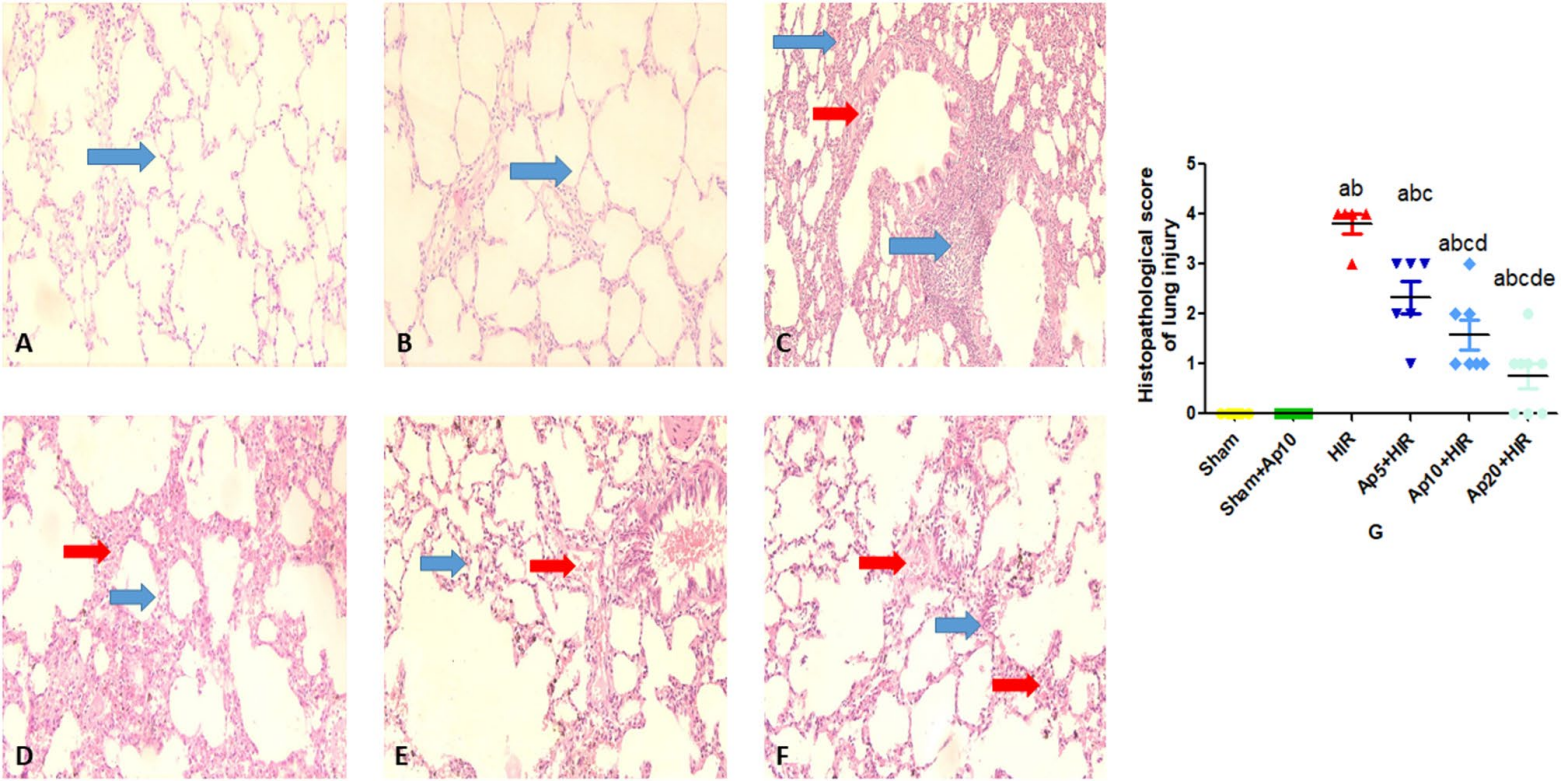
Photomicrographs of rats’ lung tissues in hepatic ischemia reperfusion- induced lung injury in rats: Sham group (**A**), Sham + Ap10 group (**B**), HIR group (**C**), Ap5 + HIR group (**D**), the Ap10 + HIR group (**E**), the Ap20 + HIR group (**F**). Slides of both Sham and Sham-Ap10 groups showed normal lung architecture composed of normal lung tissue composed of thin-walled alveoli and bronchioles (blue arrows) with absence of any inflammation. Slides of HIR group showed marked alveolar edema, hemorrhage (red arrow), thickening and inflammatory cells infiltrate (blue arrow) involving more than 75% of the lungs. Slides of Ap5 + HIR group showed moderate alveolar edema, hemorrhage (red arrow), thickening and inflammatory cells infiltrate (blue arrow) involving 50–75% of the lungs. Slides of Ap10 + HIR group showed mild alveolar edema, hemorrhage (red arrow), thickening and inflammatory cells infiltrate (blue arrow) involving 25–50% of the lungs. Ap20 + HIR group showed minimal alveolar edema, hemorrhage (red arrow), thickening and inflammatory cells infiltrate (blue arrow) involving less than 25% of the lungs. X200. The histopathological score of lung injury in the studied groups (G) represent the mean ± SEM (n = 5–8). Results are considered significantly different when *p* < 0.05. ^a^significantly different from sham group. ^b^significantly different from Sham + Ap10 group. ^c^significantly different from HIR group. ^d^significantly different from Ap5 + HIR group. ^e^significantly different from Ap10 + HIR group. (Ap5: Aprepitant 5 mg/kg; Ap10: Aprepitant 10 mg/kg; Ap20: Aprepitant 20 mg/kg; HIR: Hepatic ischemia reperfusion).

Slides of both Sham (Fig. 2A) and Sham + Ap10 (Fig. 2B) groups showed normal lung architecture composed of normal lung tissue composed of thin-walled alveoli and bronchioles (blue arrows) with absence of any inflammation. Slides of HIR group (Fig. 2C) showed marked alveolar edema, hemorrhage (red arrow), thickening and inflammatory cells infiltrate (blue arrow) involving more than 75% of the lungs. Slides of Ap5 + HIR group (Fig. 2D) showed moderate alveolar edema, hemorrhage (red arrow), thickening and inflammatory cells infiltrate (blue arrow) involving 50–75% of the lungs. Slides of Ap10 + HIR group (Fig. 2E) showed mild alveolar edema, hemorrhage (red arrow), thickening and inflammatory cells infiltrate (blue arrow) involving 25–50% of the lungs. Ap20 + HIR group (Fig. 2F) showed minimal alveolar edema, hemorrhage (red arrow), thickening and inflammatory cells infiltrate (blue arrow) involving less than 25% of the lungs. The histopathological score of lung injury in the studied groups was evaluated (Fig. 2G).

### Effect of different doses of aprepitant on lung IL-1β immunohistochemical expression in hepatic ischemia reperfusion-induced acute lung injury in rats

**Fig. 3:**
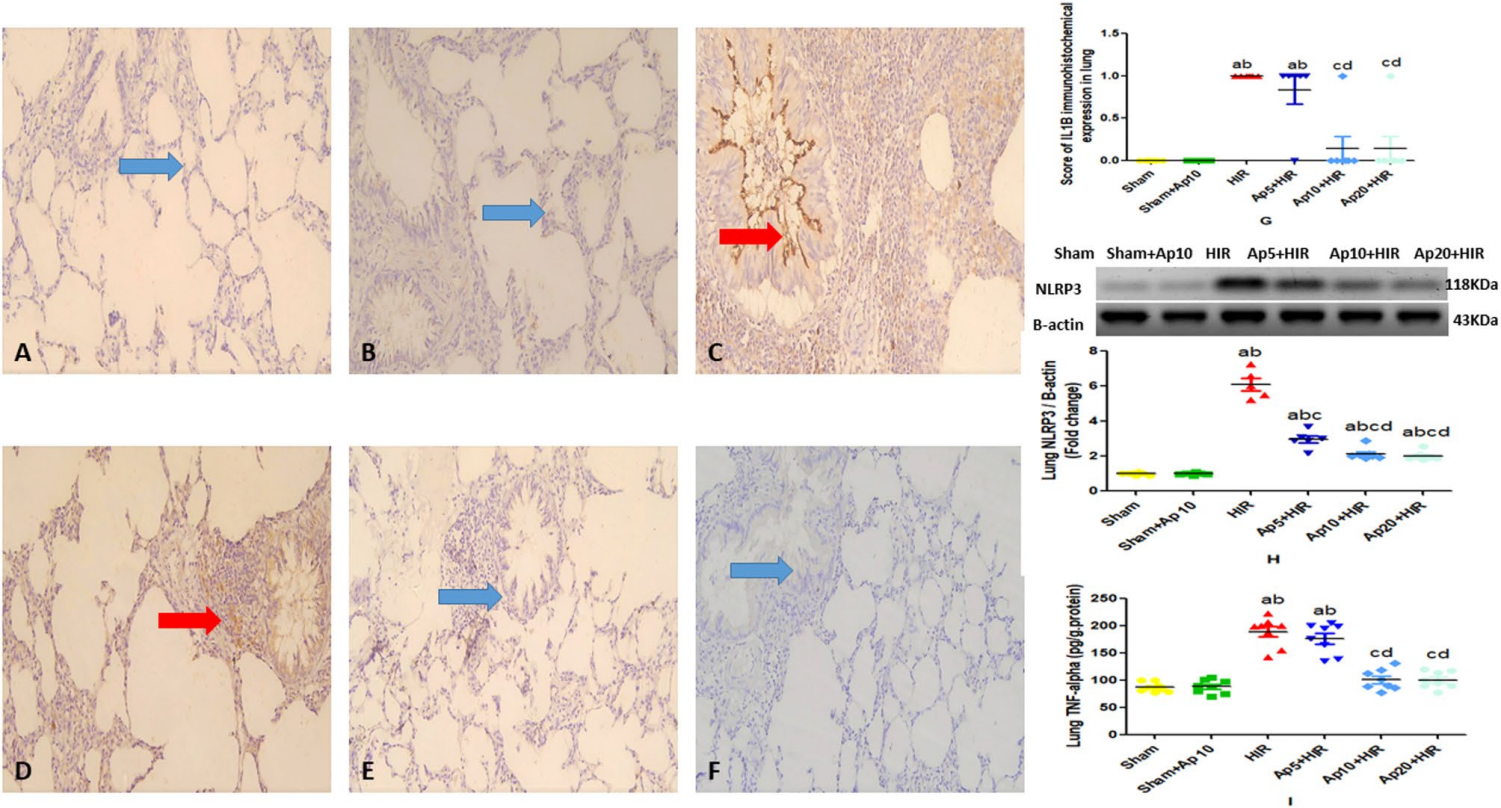
Effect of different doses of aprepitant on lung inflammatory parameters in hepatic ischemia reperfusion-induced lung injury in rats. Immunohistochemical expression of lung IL-1β in hepatic ischemia reperfusion induced lung injury in rats: Sham group (**A**), the Sham-Ap10 group (**B**), HIR group (**C**), Ap5 + HIR group (**D**), Ap10 + HIR group (**E**), Ap20 + HIR group (**F**). The Ap10 + HIR and Ap20 + HIR group showed a significant decrease in the lung IL-1β immune staining (blue arrows) in comparison to HIR group (red arrow) that showed a significant increase in the number of IL-1β immunoreactive lung tissue, there is no significant difference between the HIR group and Ap5 + HIR group (red arrow), and no significant difference between the Ap10 + HIR and Ap20 + HIR groups in comparison to the Sham and Sham + Ap10 groups (blue arrows). X200. The score of IL-1β immunohistochemical expression in lung in the studied groups (**G**) represent the mean ± SEM (n = 5–8). Results are considered significantly different when *p* < 0.05. ^a^significantly different from sham group. ^b^significantly different from Sham + Ap10 group. ^c^significantly different from HIR group. ^d^significantly different from Ap5 + HIR group. (Ap5: Aprepitant 5 mg/kg; Ap10: Aprepitant 10 mg/kg; Ap20: Aprepitant 20 mg/kg; HIR: Hepatic ischemia reperfusion; IL-1β: Interleukin—1β). Effect of different doses of aprepitant on lung NLRP3 inflammosome expression and TNF-α level in hepatic ischemia reperfusion-induced lung injury in rats were represented in Fig. 3H,I, respectively. Values represent the mean ± SEM (n = 5–8). Results are considered significantly different when *p* < 0.05. ^a^significantly different from sham group. ^b^significantly different from Sham + Ap10 group. ^c^significantly different from HIR group. ^d^significantly different from Ap5 + HIR group. ^e^significantly different from Ap10 + HIR group. (Ap5: Aprepitant 5 mg/kg; Ap10: Aprepitant 10 mg/kg; Ap20: Aprepitant 20 mg/kg; HIR: Hepatic ischemia reperfusion; NLRP-3: Nucleotide-binding oligomerization domain—like receptors protein 3; TNF-α: tumor necrosis factor alpha).

The Ap10 + HIR and Ap20 + HIR group showed a significant decrease in the lung IL-1β immune staining in comparison to HIR group that showed a significant increase in the number of IL-1β immunoreactive lung tissue, there is no significant difference between the HIR group and Ap5 + HIR group, and no significant difference between the Ap10 + HIR and Ap20 + HIR groups in comparison to the Sham and Sham + Ap10 groups (Fig. 3).The score of IL-1β immunohistochemical expression in lung in the studied groups.

## Discussion

HIR injury is a critical clinical condition that occurs during various clinical surgeries such as liver transplantation, liver resection, and hemorrhagic shock. This can result in tissue damage, graft dysfunction or rejection and liver cell failure^24,25^. Moreover, it often leads not only to damage in the liver, but also to other remote organs injury^26^. Remote organ injury significantly worsens patient outcomes and requires effective and preventative strategies to prevent its occurrence. Medication that scavenges ROS and reduces inflammation may be helpful, despite limited data on approaches to reduce remote organ damage^16^. ALI and the more severe form; acute respiratory distress syndrome (ARDS), are common complications of liver transplantation and major liver surgery, and thereby significantly contributed to perioperative morbidity and mortality^27^.

In our study, HIR resulted in significant oxidative stress in lung tissue, as evidenced by an elevation of lung MDA and significant decrease in TAC levels. Excess MDA produced by tissue injury can alter the structure of molecules by combining with free amino group of protein leading to the formation of MDA modified protein adducts that render them immunogenic^28^. Similarly, the TAC and GSH values reflect the organism’s antioxidative status including the synergic and redox interactions among various substances in the biological fluids^29^.

Oxidative stress is known to play a central role in ALI. Extensive ROS overproduction occurs in ALI, overwhelming endogenous antioxidants and allowing oxidative cell injury. The majority of experimental models of lung damage include evidence of oxidative stress, which is supported by investigations on patients with ALI. When lung damage occurs, neutrophil activation causes an overabundance of oxygen radicals to be produced, which changes lung function metrics^30^.

It is noteworthy that Ap antioxidant activity could be attributed to the scavenging of oxidative radicals generated by HIR. This was in agreement with the aforecited studies by Kose et al.^15^, Hafez et al.^16^, Mohamed et al.^17^, and Mohyeldin et al.^11^. who proved that Ap has antioxidant characters. This was observed in the present study in the co-administrated Ap in different doses with HIR groups as Ap improved significantly the oxidative stress parameters in dose dependent manner.

Mechanistically, the lungs are the first capillary bed that is reached by the blood after leaving the hepatic circulation, one proposed mechanism is that ALI may be induced by proinflammatory mediators released from the injured liver into system circulation. Also, activation of many intracellular signaling pathways by oxidative stress leads to the up-regulation of pro-inflammatory cytokine production such as TNF-α. Flow affection and proinflammatory release in HIR can cause mitochondrial swelling leading to cytochrome-c release which initiates caspase cascade activation resulting in mitochondrial damage and initiating the process of apoptosis^31^.

Regarding the apoptotic marker caspase-3, our study demonstrated that lung caspase-3 was upregulated by HIR and this was consistent with a previous study that revealed a similar finding in an acute renal injury model in rats^32^. Notably, caspase-3 was upregulated in rats with acute lung injury^33^.

Pretreatment with Ap in different doses resulted in a significant reduction in lung caspase-3 in the current result. Thus, in accordance with the previously cited study of Hafez and coauthors^16^ who demonstrated the anti-apoptotic role of Ap in diclofenac- induced renal toxicity in a rat model.

Interestingly, Kupffer cells (KCs) express NLRP3 which undergoes activation by various stimuli. Activation of KCs plays a significant role in regulating inflammation, through activation of the inflammatory caspase protein kinase (Caspase-1), causing an overproduction of the inflammatory cytokines e.g. IL-1β^34^. These bioactive cytokines are essential for initiating and intensifying the inflammatory reactions that occur during ALI^35^. Furthermore, inhibition of NLRP3 activation leads to a reduction in the ROS level and the degree of liver injury in HIR^34,36,37^. Targeted suppression of NLRP3 inflammasome and related signaling pathways represents a new direction in ALI prevention and treatment research^9^.

Notably, the increase of NLRP3, TNF-α and IL-1β in remote organs following HIR was previously demonstrated in kidney^38^. Other studies have also showed a similar increase in lung NLRP3 and IL-1β^35,39^. These earlier studies were in accordance with our current findings which demonstrated that HIR produced an altitude in lung NLRP3 Western expression, TNF-α level and IL-1β immuno-expression. Contrary, AP-treated groups showed significant decrease in lung NLRP3 Western expression, TNF-α level and IL-1β immuno-expression.

It has been previously shown that Ap has anti-inflammatory effects and could block the NK-1R pathway in macrophages, suppressing inflammation^40^. Ap can suppress the expression of chemokines and cytokines in rheumatoid arthritis, hepatotoxicity and nephrotoxicity^13,41^. Additionally, it has anti-inflammatory effect by reducing TNF-α in lung tissue^15^.

Our current study showed that HIR resulted in marked alveolar edema, hemorrhage, thickening and inflammatory cell infiltrate involving more than 75% of the lungs. These results were in accordance with previous studies^12,42,43^.

Administration of Ap revealed a dose dependent improvement in histopathological picture of lung injury in the form of reduction of the degree and extent of alveolar edema, hemorrhage, thickening and inflammatory cells infiltrate. This finding was in consistent with a previous study of Mao et al.^44^ as well as with Kose and colleagues^15^ who demonstrated that the administration of different doses of Ap resulted in histopathological improvement of sepsis induced lung injury due to its anti-inflammatory and antioxidant properties**.**

### Limitations of the study

It is worthy to note that the result of the current study is limited as it lacks evaluation of Ap in a curative model. Ap should be used in 20 mg/kg as sham in our study. Therefore, we strongly recommend further studies for evaluation of the limited points.

## Conclusion

The current study provides experimental evidence that Ap -in different doses- as a potential pharmacological tool for ameliorating ALI induced via HIR through its anti-inflammatory, antioxidant, anti-apoptotic properties, as well as its modulation of the NLRP3 inflammasome/ IL-1β pathway that could reveal other potential drugs in further research studies.

## Supplementary Information

Below is the link to the electronic supplementary material.

Supplementary Material 1.

## Data Availability

The data sets used and/or analyzed during the current study are available from the corresponding author on reasonable request.
