# Supplementary material for: Aprepitant alleviates acute lung injury in a rat model of hepatic ischemia–reperfusion via NLRP3/IL-1β signaling pathway
Source: Sci Rep. 2025 Nov 18;15:40358. doi: 10.1038/s41598-025-25930-4 (PMC12627803; doi:10.1038/s41598-025-25930-4)
Supplement: Supplementary file 1 — Supplementary Material 1. [file 41598_2025_25930_MOESM1_ESM.pdf]

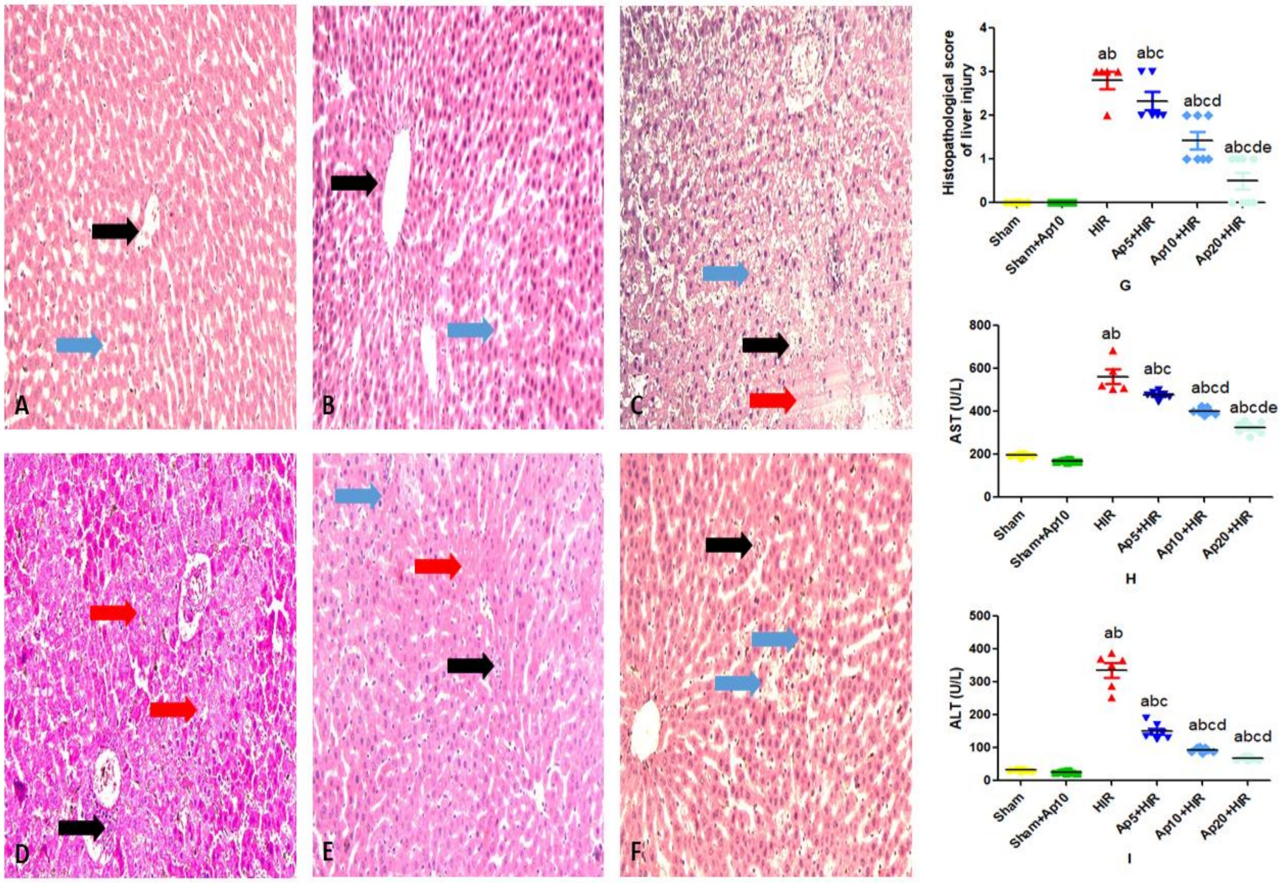

Figure (S1): Photomicrographs of rats' hepatic tissues with liver enzymes in HIR induced liver injury in rats: Sham group (A), Sham-Ap10 group (B), HIR group (C), Ap5+HIR group (D), the Ap10+HIR group (E), the Ap20+HIR group (F).

Slides of both Sham and Sham-Ap10 groups showed normal liver architecture composed of normal central vein (black arrows) lined by endothelial cells with normal polygonal hepatocytes (blue arrows) with eosinophilic cytoplasm arranged around it with absence of any inflammation. Slides of HIR group showed marked centrilobular necrosis (red arrow), hepatocyte degeneration (blue arrow) and inflammatory cells infiltrate (black arrow) (total score 9). Slides of Ap5+HIR group showed moderate hepatocyte necrosis (red arrow), degeneration and inflammatory cells infiltrate (black arrow) (total score 6). Slides of Ap10+HIR group showed mild hepatocyte necrosis (red arrow), degeneration (blue arrow) and inflammatory cells infiltrate (black arrow)

(total score 3). Ap20+HIR group showed mild degeneration (blue arrow) and inflammatory cells infiltrate (black arrow) with no necrosis (total score 2). X200

The histopathological score of liver injury in the studied groups represent the mean ± SEM (n=5-8). Results are considered significantly different when $p < 0.05$. [a] significantly different from sham group. [b] significantly different from Sham-Ap10 group. [c] significantly different from HIR group. [d] significantly different from Ap5+HIR group. [e] significantly different from Ap10+HIR group. (Ap5: Aprepitant 5mg/kg; Ap10: Aprepitant 10mg/kg; Ap20: Aprepitant 20mg/kg; HIR: Hepatic ischemia reperfusion).

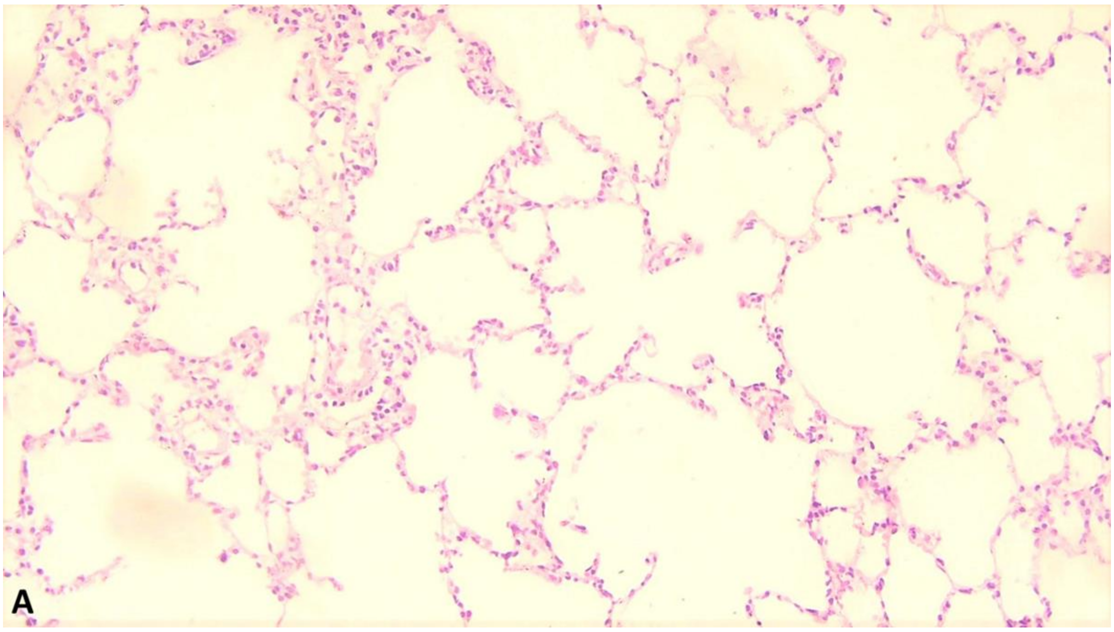

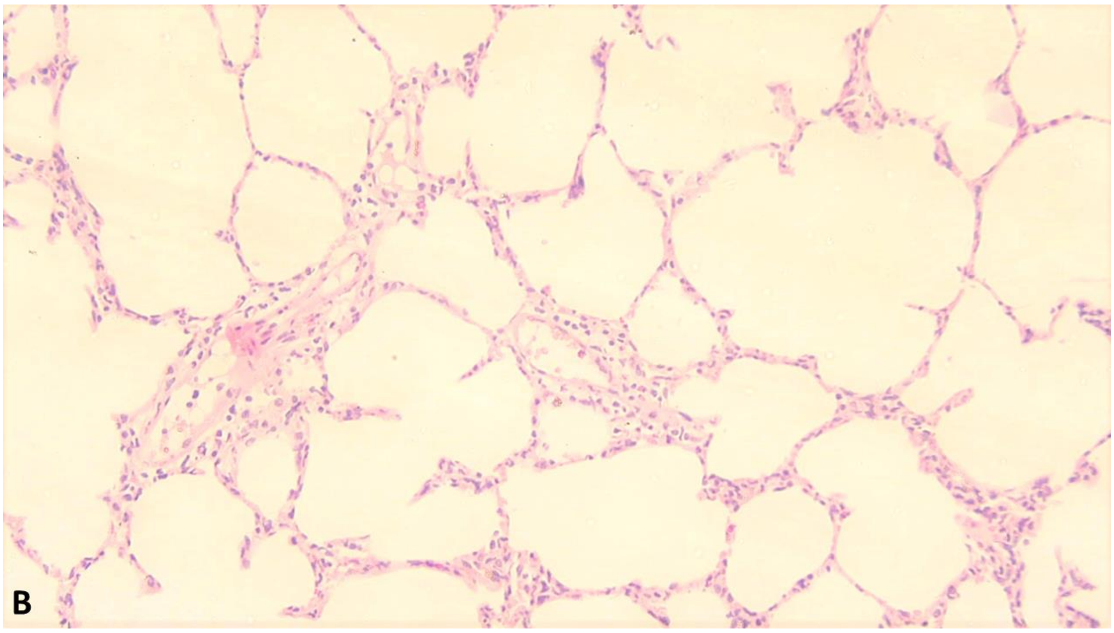
B

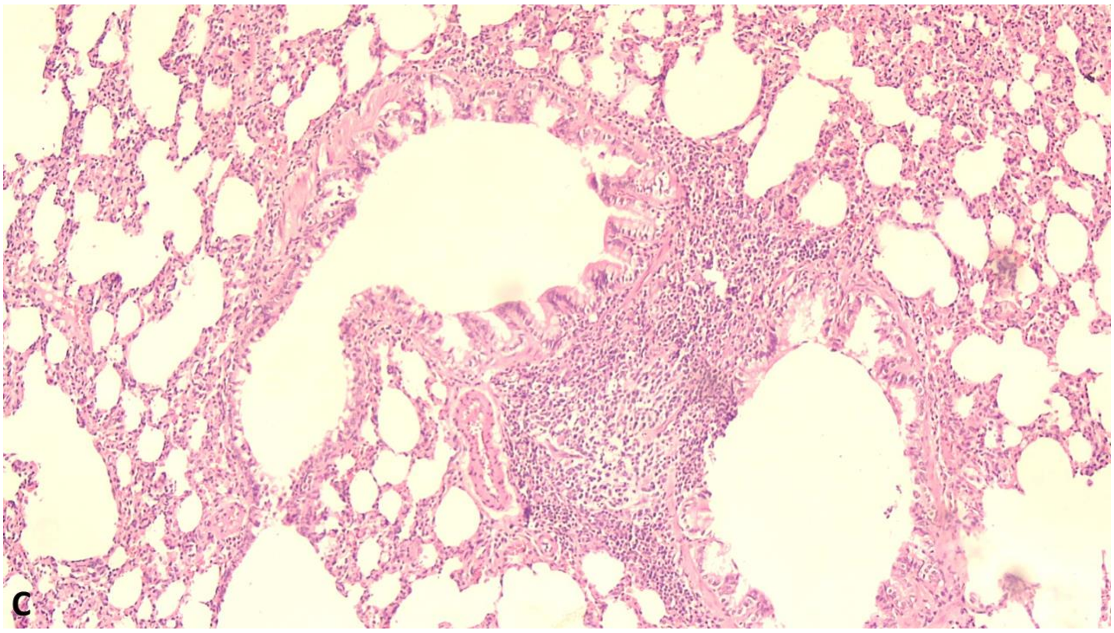
C

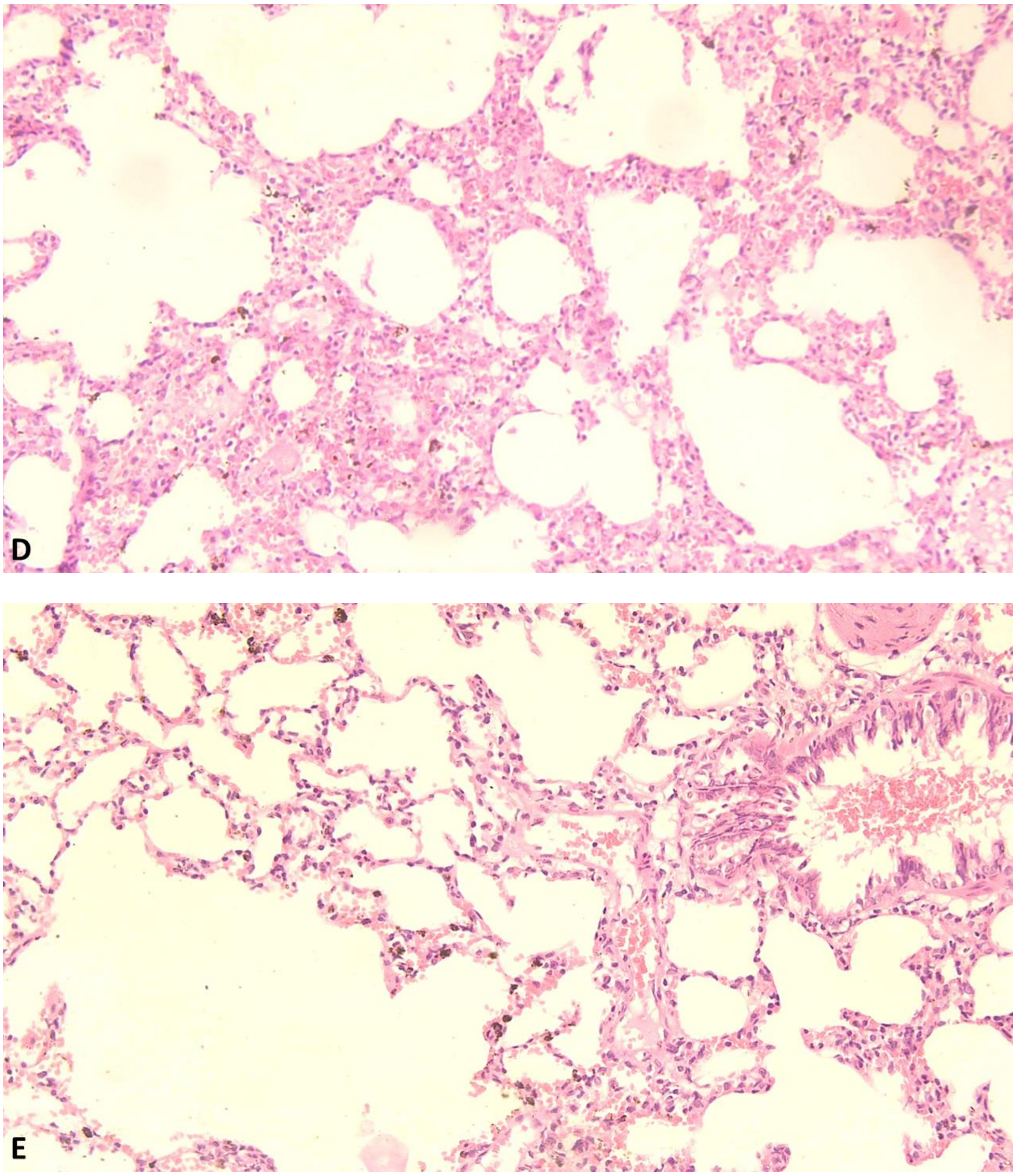
D
E

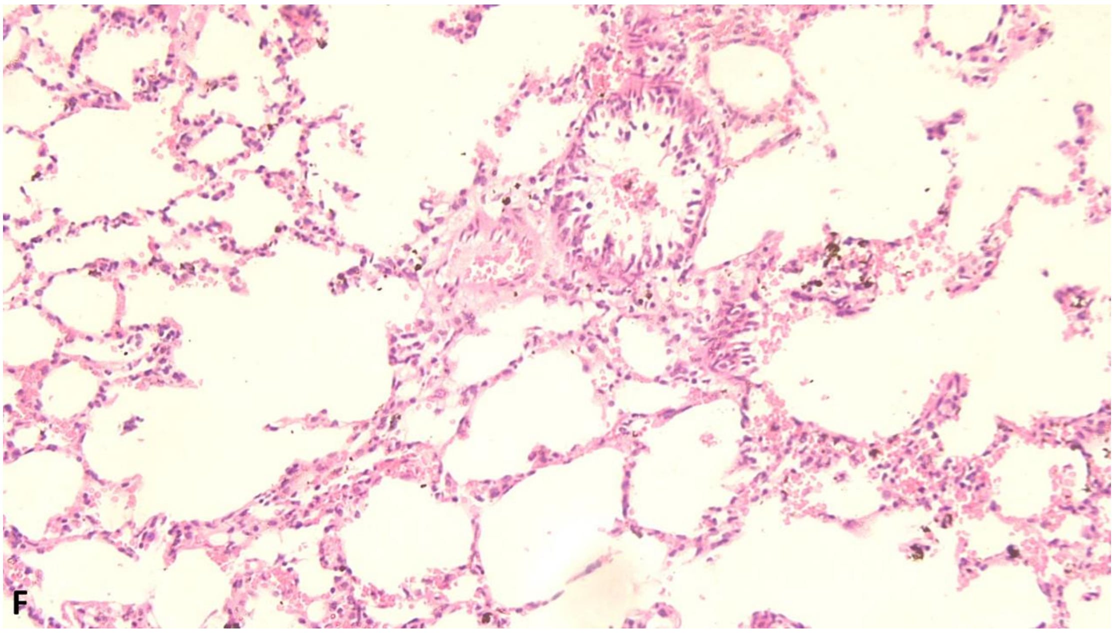

Figure (S2): Photomicrographs of rats' lung tissues in hepatic ischemia reperfusion- induced lung injury in rats: Sham group (A), Sham-Ap10 group (B), HIR group (C), Ap5+HIR group (D), the Ap10+HIR group (E), the Ap20+HIR group (F). Slides of both Sham and Sham-Ap10 groups showed normal lung architecture composed of normal lung tissue composed of thin-walled alveoli and bronchioles (blue arrows) with absence of any inflammation. Slides of HIR group showed marked alveolar edema, hemorrhage (red arrow), thickening and inflammatory cells infiltrate (blue arrow) involving more than 75% of the lungs. Slides of Ap5+HIR group showed moderate alveolar edema, hemorrhage (red arrow), thickening and inflammatory cells infiltrate (blue arrow) involving 50-75% of the lungs. Slides of Ap10+HIR group showed mild alveolar edema, hemorrhage (red arrow), thickening and inflammatory cells infiltrate (blue arrow) involving 25-50% of the lungs. Ap20+HIR group showed minimal alveolar edema, hemorrhage (red arrow), thickening and inflammatory cells infiltrate (blue arrow) involving less than 25% of the lungs. X200.

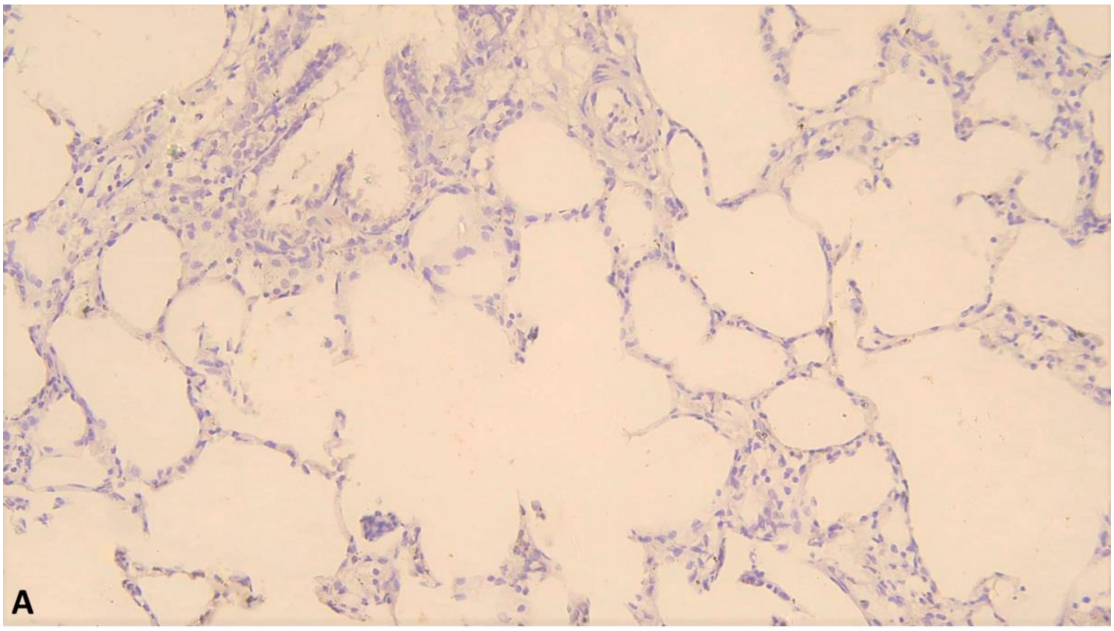
A

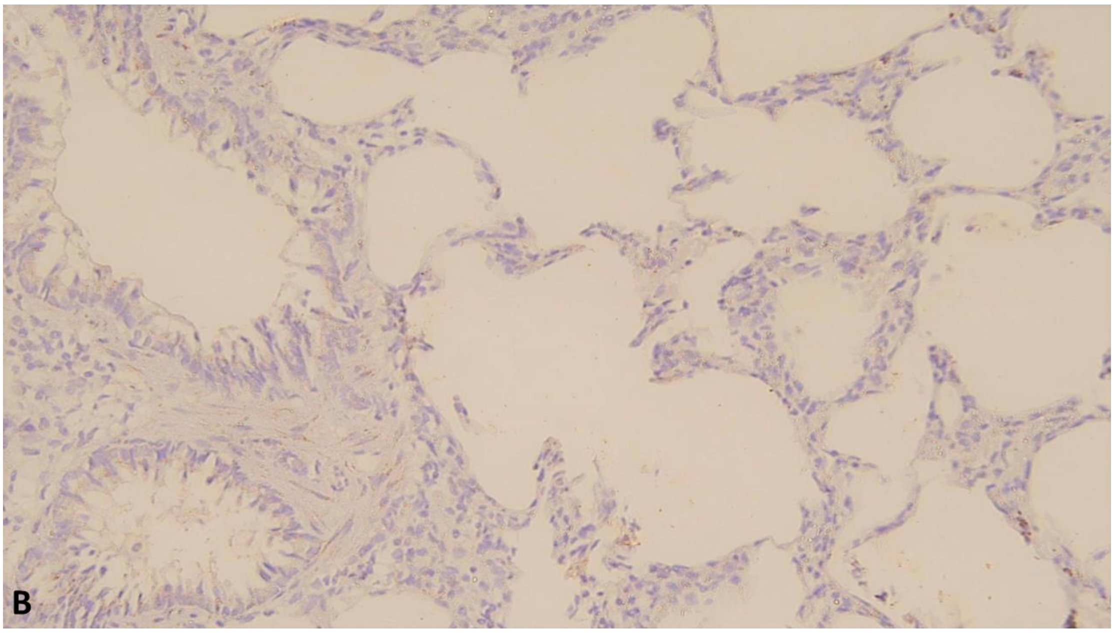
B

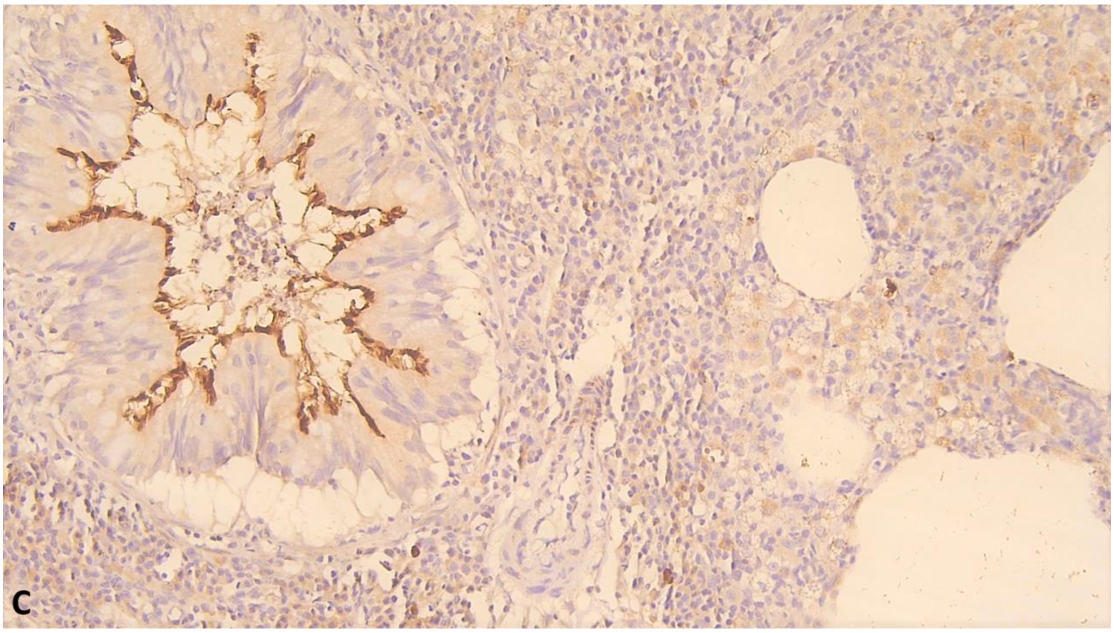

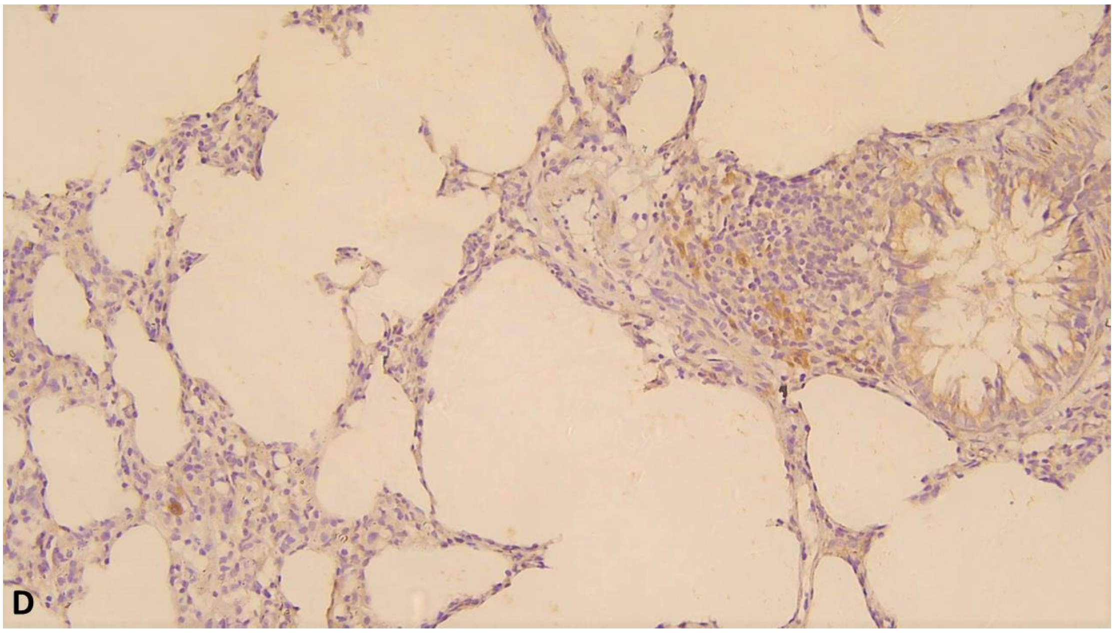

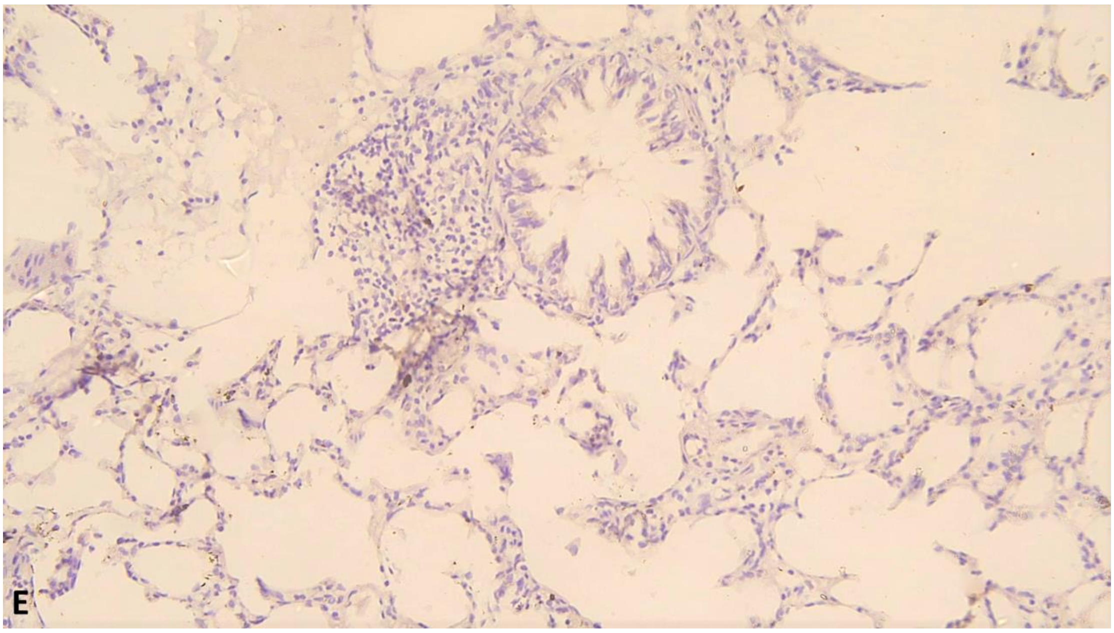

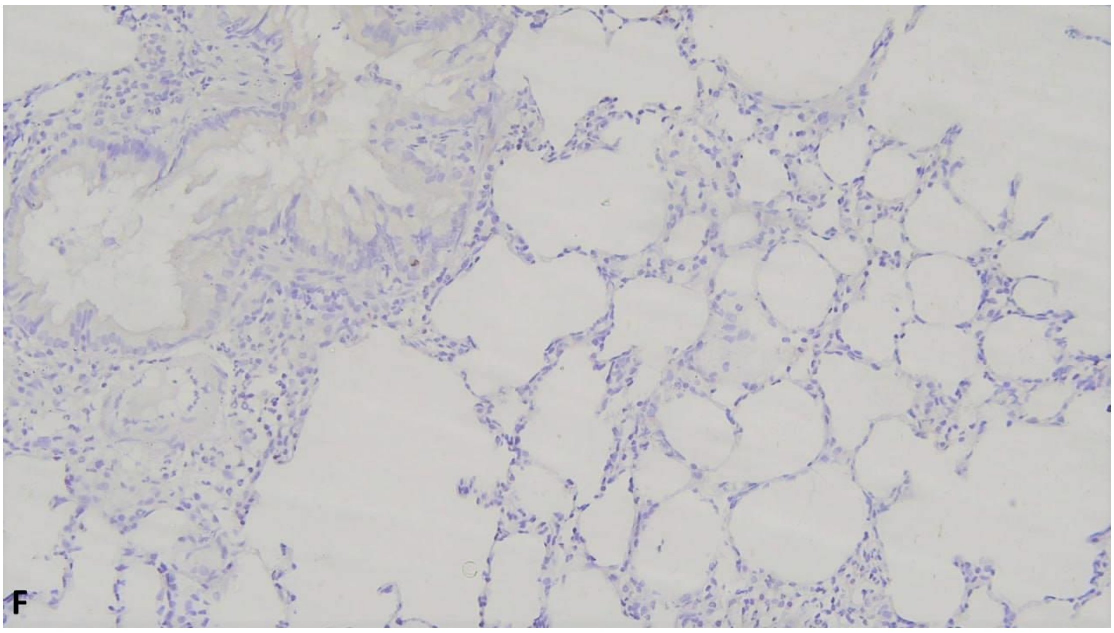

Figure (S3): Immunohistochemical expression of lung IL-1β in hepatic ischemia reperfusion induced lung injury in rats: Sham group (A), the Sham-Ap10 group (B), HIR group (C), Ap5+HIR group (D), Ap10+HIR group (E), Ap20+HIR group (F). The Ap10+HIR and Ap20+HIR group showed a significant decrease in the

lung IL-1β immune staining (blue arrows) in comparison to HIR group (red arrow) that showed a significant increase in the number of IL-1β immunoreactive lung tissue, there is no significant difference between the HIR group and Ap5+HIR group (red arrow), and no significant difference between the Ap10+HIR and Ap20+HIR groups in comparison to the Sham and Sham-Ap10 groups (blue arrows). X200

Lung NLRP3

Repeat 1

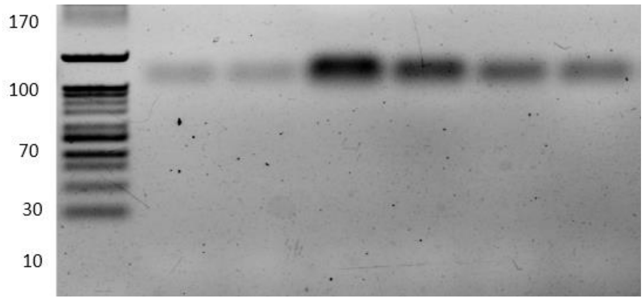

NLRP3 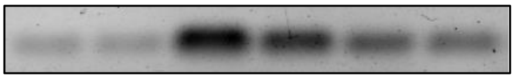 118 KDa

Repeat 2

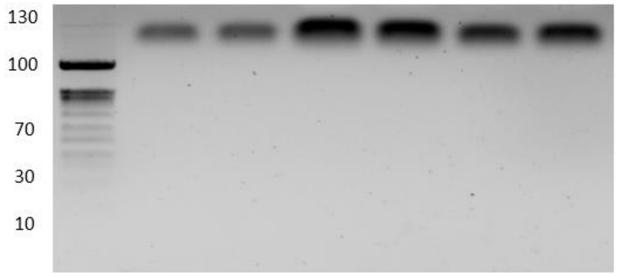

NLRP3 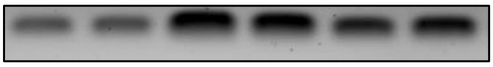 118 KDa

Repeat 3

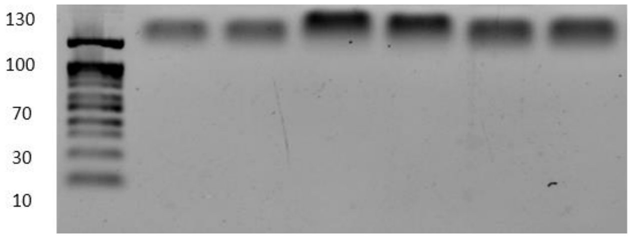

NLRP3 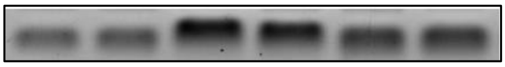 118 KDa

Lung B-actin

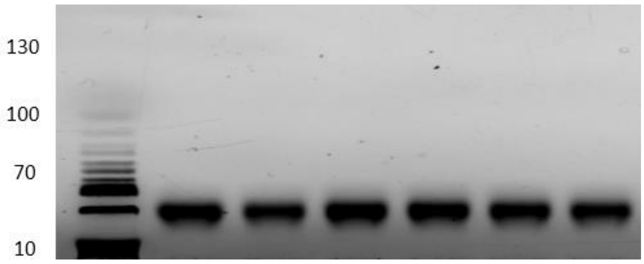

B-actin 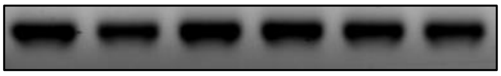 43 KDa

Lung c-Caspase 3

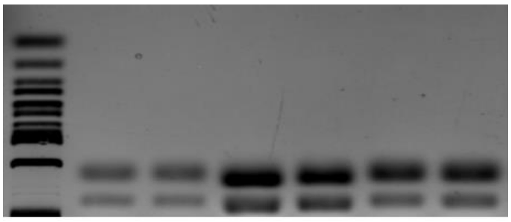

c-Caspase 3 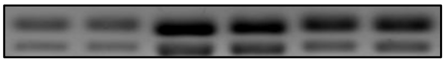 17,19 KDa

Lung B-actin

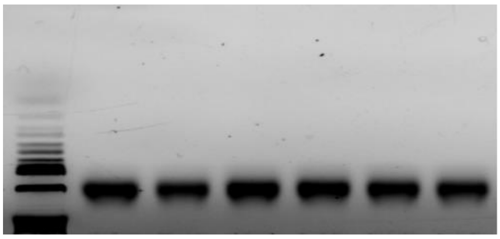

B-actin 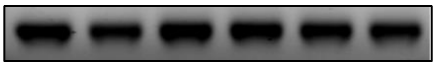 43 KDa
